# Novel Respiratory Breath Hold Index: A Predictor for Successful Extubation in Mechanically Ventilated Patients, a Prospective Cohort Study

**DOI:** 10.3390/life15101541

**Published:** 2025-10-01

**Authors:** Arie Soroksky, Gingy Ronen Balmor, Riziny Nugzar, Adam Lee Goldstein, Emad Tayem, Ori Galante, Milena Tocut

**Affiliations:** 1 Intensive Care Department, E. Wolfson Medical Center, Holon 58100, Israel; 2 Faculty of Medicine, Tel Aviv 69978, Israel; 3 Anesthesia Department, E. Wolfson Medical Center, Holon 58100, Israel; 4 Surgical Department A Trauma Division, E. Wolfson Medical Center, Holon 58100, Israel; 5 Medical Intensive Care Unit, Soroka University Medical Center, Be’er Sheva 84101, Israel; 6 Faculty of Health Sciences, Ben-Gurion University of the Negev, Be’er Sheva 84101, Israel; 7 Internal Medicine C Department, Wolfson Medical Center, Holon 58100, Israel

**Keywords:** extubation, liberation from mechanical ventilation, spontaneous breathing trial, respiratory breath hold index

## Abstract

Background: Liberation from mechanical ventilation is a major objective in critically ill patients. Various criteria for extubation are used with different success rates. We developed a novel, simple bedside maneuver and index that involves measuring breath-hold duration and forced vital capacity (FVC). We named it the Respiratory Breath Hold Index (RBHI). Methods: We enrolled 225 mechanically ventilated intensive care unit (ICU) patients who were candidates for extubation. At the end of a spontan eous breathing trial (SBT), and just prior to extubation, patients were asked to hold their breath and perform a stalked FVC maneuver. The ability to perform a breath-hold maneuver and its duration were recorded and compared with a standard SBT. Results: 171 patients (76%) were successfully extubated, while 54 patients (24%) failed extubation. A successful SBT alone did not predict extubation, as 80.1% of passed SBT and 81.5% of failed SBT patients were extubated successfully (*p* = 1.00). However, a higher RBHI, together with the ability to hold breath and breath-hold duration, was highly associated with a successful extubation (*p* < 0.0001). Logistic regression analysis showed that RBHI over 3 was correlated with higher rates of successful extubation (OR 4.252, *p* < 0.001). Overall, 89% of patients who passed SBT and were able to hold breath were successfully extubated. (*p* < 0.0001). Whereas, among patients who passed SBT but failed to hold breath, only 24% were successfully extubated (*p* < 0.0001). Conclusion: Higher RBHI, together with the ability to hold a breath just prior to extubation in mechanically ventilated patients, is more sensitive and specific, and may be superior to standard SBT in predicting a successful extubation.

## 1. Introduction

Predicting the accurate time for liberation from mechanical ventilation has great consequences for mechanically ventilated patients [1]. Patients who are extubated too early are at increased risk for reintubation and worse outcomes [2,3]. Reintubation in itself is associated with increased risk for ventilation-acquired pneumonia (VAP) [4,5,6], length of ICU and hospital stay [7,8], and possibly with an increased mortality [8,9,10,11].

Gaëtan Béduneau and colleagues reported that a prolonged duration of mechanical ventilation, intensive care unit (ICU) stay, and mortality significantly increased mechanical ventilation duration with unsuccessful attempts of extubation. The unadjusted risk of dying was 19% after the first extubation and increased to 37% ten days after [12]. Prolongation of mechanical ventilation is also associated with worsened survival [13]. Failure to identify the readiness of a patient for extubation as soon as the patient is actually ready for liberation from mechanical ventilation may prolong mechanical ventilation unnecessarily, resulting in increased risk of complications, such as hospital-acquired infections and death [13]. Therefore, making the right decision at the right time has immense consequences on the outcome of mechanically ventilated patients.

Many criteria for extubation have been developed and used over the last years [14,15,16]. All are trying to aid us in predicting and choosing the eligible patients and adequate time for ensuring a successful liberation from mechanical ventilation.

So far, the available predicting criteria have limited sensitivity and specificity [17,18,19,20], including the commonly used rapid shallow breathing index [21,22]. Using the currently acceptable criteria to identify the patients that can be weaned from mechanical ventilation has an approximately 15 to 20% chance of weaning failure resulting in reintubation [23,24,25].

We sought to develop a simple bedside parameter, the ability to perform a breath hold, along with an index that combines the product of breath hold duration with stalked forced vital capacity (FVC), that could aid in predicting a successful extubation. We named it the Respiratory Breath Hold Index (RBHI).

## 2. Materials and Methods

This prospective cohort study was performed in a single 16-bed university-affiliated ICU at Wolfson Medical Center, Israel and was approved by the hospital’s Helsinki institutional review board (registration number 0164-11-WOMC). A waiver for informed consent was granted to record all breathing patterns and related data in all recruited patients.

A total of 225 consecutive patients who were undergoing weaning from mechanical ventilation were recruited. Only those who were actually extubated were included in the study. Unconscious patients with a Glasgow coma scale (GCS) less than 10, or patients who were not cooperating with simple commands, were not recruited.

Daily screening of readiness for weaning from mechanical ventilation was performed by the attending physicians, assessing 6 criteria:

The ratio of partial pressure of oxygen in arterial blood to the fraction of inspiratory oxygen concentration (PaO_2_/FiO_2_ ratio) > 150, positive end-expiratory pressure (PEEP) 5 to 8 cmH_2_O, adequate cough reflex during tracheal suction, respiratory rate to tidal volume ratio (f/V) of less than 105, low dose or no administration of sedatives, and a stable respiratory and hemodynamic state.

Patients who met all 6 criteria and were found to be suitable for mechanical ventilation weaning were subjected to a spontaneous breathing trial (SBT). The duration of the SBT trial was 30 to 120 min and was determined by the attending physician. All patients were subjected to the same settings, continuous positive airway pressure (CPAP) mode with PEEP of 5 cm H_2_O and inspiratory pressure augmentation with pressure support of 7 cm H_2_O. We did not perform hyperventilation, nor did we preoxygenate patients before asking them to breathe hold.

A failure of SBT was defined by the following criteria: SpO_2_ lower than 90% with an FiO_2_ higher than 50%, increase in PCO_2_ to more than 60 mmHg, or by more than 20 mmHg from baseline, exhaled tidal volume < 5 mL/kg ideal body weight, heart rate more than 140 beats/min or increase in heart rate by 20% from baseline for more than 5 min, systolic blood pressure less than 90 mmHg systolic or more than 200 mmHg systolic for more than 5 min, new onset anxiety, decreased level of consciousness, tachypnea with respiratory rate of more than 35 breathes/min for more than 5 min and new onset of use of accessory muscles of respiration.

A successful SBT led to immediate extubation. However, the final decision on extubation was made by the attending physician, who, based on clinical judgement, was allowed to abort SBT or to proceed to extubation even in patients who failed SBT. Since the analysis was performed prospectively, the attending physician had no knowledge of the final results of the RBHI test.

At the end of each SBT trial and just prior to extubation, patients were asked to perform a respiratory hold maneuver by simply holding their breath. The ability to perform breath holding and breath hold duration was recorded. FVC was recorded immediately after a holding maneuver by asking patients to perform a maximal forced exhalation. The product of multiplying FVC and the duration of breath holding in seconds was computed as the RBHI.

The statistical analysis aimed to evaluate the efficacy of the novel score, RBHI, in comparison to the standard SBT. Continuous variables were summarized using median and interquartile range (IQR), with intergroup comparisons conducted through the Kruskal–Wallis test. For categorical variables, counts and percentages were employed for summarization, and group comparisons were made using Fisher’s exact test.

To assess the discriminative ability of the scores in predicting reintubation within 48 h, a receiver operating characteristic (ROC) model was applied. This model was instrumental in determining the optimal threshold for identifying reintubation within 48 h.

Logistic regression models were employed to ascertain factors influencing the odds of reintubation within 48 h, with adjustments made for age, sequential organ failure assessment (SOFA) and APACHE II scores. The significance level was set at *p* < 0.05, employing a two-sided test. All analyses were conducted using R version 4.3.2 (R Foundation for Statistical Computing, Vienna, Austria).

## 3. Results

The study included 225 patients. One hundred eighty-one patients passed SBT successfully (80%). Among those 181 patients who passed SBT, 144 patients succeeded in holding their breath (80%), and only 16 patients were reintubated (11%), while 37 patients did not succeed in holding their breath (20%), and 28 patients were reintubated (76%).

In comparison, 44 patients (20%) with successful extubation failed SBT prior to extubation. Despite failing the SBT trial, 32 patients (72%) managed to hold their breath, and only two patients were reintubated (6%). Thus, despite failure in SBT, most of those patients who were able to hold breath were extubated successfully.

**Figure 1:**
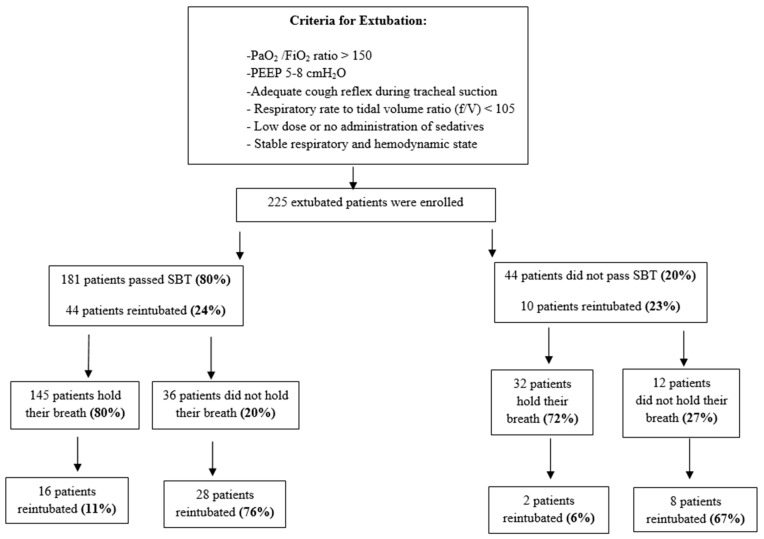
Spontaneous breathing test, breath-hold test and outcomes of extubated patients. **SBT**—Spontaneous breathing trial, **PEEP**—Positive end-expiratory pressure, **PaO_2_/FiO_2_**—The ratio of partial pressure of oxygen in arterial blood to the fraction of inspiratory oxygen concentration.

On the other hand, 12 (27%) patients did not manage to hold their breath, and 8 patients were reintubated (67%). Thus, failure of both SBT and breath holding carries a 79% chance of being reintubated (Figure 1).

The study included 126 (56%) males and 99 (44%) females. The mean age was 67 ± 17 years old. Mean BMI was 28 ± 7. One hundred seventy-one patients (76%) were successfully extubated, while 54 patients (24%) failed extubation. The use of NIV after extubation, ventilator-free days, ICU length of stay, ICU and hospital mortality were significantly higher (*p* < 0.0001) in patients who failed weaning from mechanical ventilation. Out of the 54 patients who failed extubation, 16 patients (30%) had tracheostomy versus none among the 171 patients who were successfully extubated (*p* < 0.0001, Table 1).

Respiratory parameters such as RBHI, the ability to hold a breath and breath-hold duration were significantly different (*p* < 0.0001) between patients who were successfully weaned from mechanical ventilation and those who were not (Table 2).

Patients who passed SBT were divided into two groups: patients who were able to hold breath (n = 145) and patients who were not able to hold breath (n = 36). Among patients who passed SBT and were able to hold breath, 129 patients (89%) were successfully extubated, ICU and hospital length of stay were shorter, and overall hospital mortality was lower when compared to patients who passed SBT but did not hold breath. The results were statistically significant (*p* < 0.0001, Table 3).

Table 4 presents the predictors for successful extubation that were represented by SBT (PPV 0.757, NPV 0.227), ability to hold breath (PPV 0.898, NPV 0.75), breath hold duration of at least 4.5 s (PPV 0.855, NPV 0.412), RBHI of at least 3.4 (PPV 0.853, NPV 0.433) and rapid shallow breath index (RSBI) of less than 43 (PPV 0.87, NPV 0.316).

**Figure 2:**
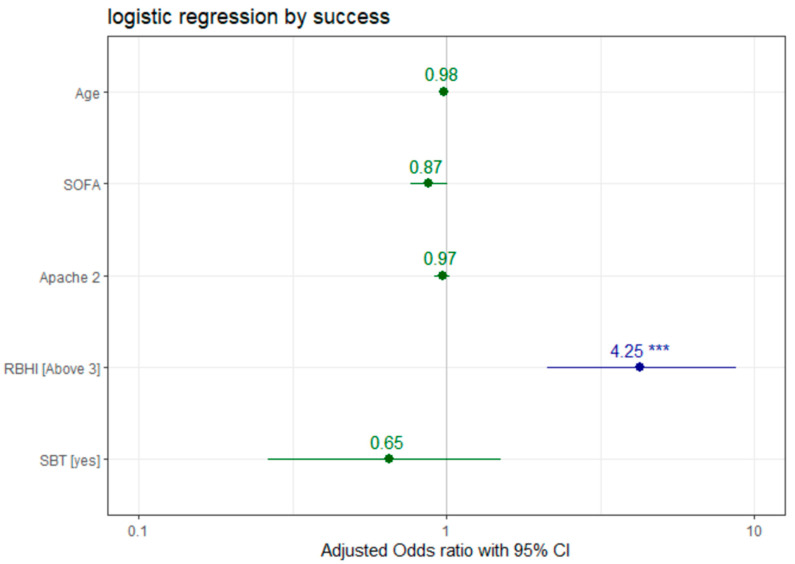
Odds ratio plot showing the relationship between different variables and success. Logistic regression models were employed to ascertain factors influencing the odds of reintubation within 48 h, with adjustments made for age, SOFA and APACHE II scores. The significance level was set at *p* < 0.05, employing a two-sided test. **SOFA**—The Sequential Organ Failure Assessment Score, **RBHI**—Respiratory Breath Holding Index, **SBT**—spontaneous breathing trial.

Logistic regression analysis (Figure 2) showed that a higher SOFA score was correlated with lower rates of successful extubation (OR 0.873, 95% CI 0.762–1.002, *p* = 0.051), while RBHI over 3 was correlated with higher rates of successful extubation (OR 4.252, 95% CI 2.1158–8.7130, *p* < 0.001). No significant correlation was found between successful extubation and other tested variables, such as age (OR 0.977, 95% CI 0.952–0.999, *p* = 0.059), APACHE II (OR 0.968, 95% CI 0.913–1.023, *p* = 0.257) score and SBT (OR 0.653, 95% CI 0.2625–1.5059, *p* = 0.335).

**Figure 3:**
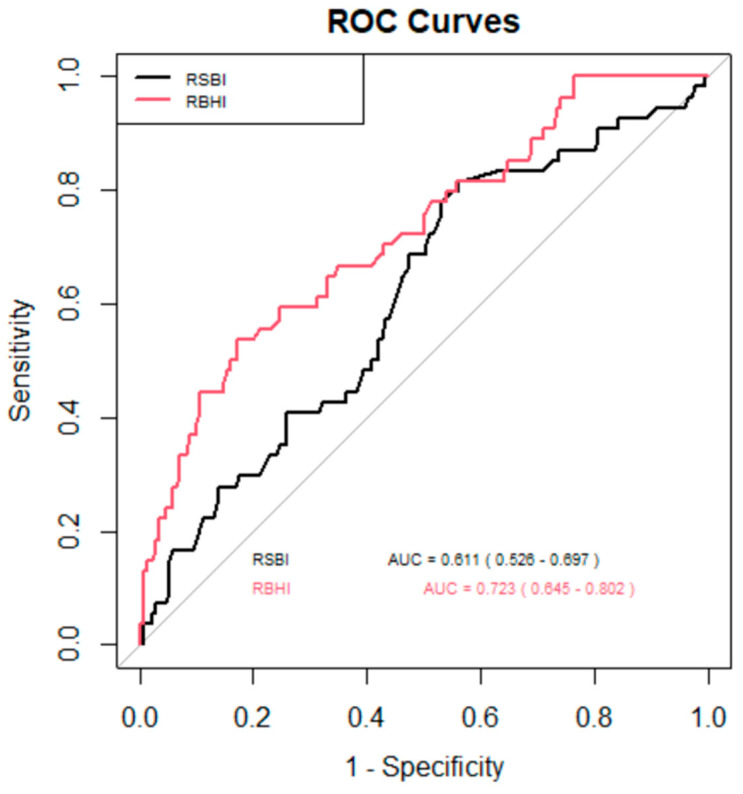
ROC analysis showing the sensitivity and specificity of the respiratory breath hold index and rapid shallow breath hold index at different cutoffs. The area under the curve (AUC) serves as a measure of each index’s ability to distinguish between successful and unsuccessful extubations. RBHI demonstrated a stronger discriminative power compared to RSBI, with an AUC of 0.723 (95% CI: 0.640.802), as compared to RSBI, which had an AUC of 0.611 (95% CI: 0.526–0.697). **RBHI**—Respiratory Breath Holding Index, **RSBI**—Rapid Shallow Breathing Index.

The ROC curve (Figure 3) was used to assess the effectiveness of two indices, RSBI and RBHI, in identifying successful extubation. The area under the curve (AUC) served as a measure of each index’s ability to distinguish between successful and unsuccessful extubations. RBHI > 3 (sensitivity 75%, specificity 59%) demonstrated a stronger discriminative power compared to rapid shallow breathing index (RSBI) (sensitivity 47%, specificity 79%), with an AUC of 0.723 (95% CI: 0.645–0.802) as compared to RSBI which had an AUC of 0.611 (95% CI: 0.526–0.697). This suggests that RBHI is more effective at predicting successful extubation outcomes than RSBI.

## 4. Discussion

The ability to hold a breath involves complex physiological processes and is influenced by conditions such as increased metabolic rate due to any cause, ongoing respiratory failure, hemodynamic instability, or any other stressful condition [26]. Increasing the metabolic rate also shortens breath-hold duration [26,27,28,29]. Exercise in the form of bicycle ergometry has been shown to at least double metabolic rate, and consequently halve breath-hold duration [27]. In comparison, decreasing the metabolic rate by cooling might prolong breath-hold duration. It has been shown that during breath-holding, lung volumes actually decrease by 200 to 500 mL each minute [30]. This is probably due to an ongoing absorption of oxygen from alveoli without replacing that volume with other gases [31]. Therefore, increasing demand for oxygen may also decrease breath-hold duration. Exercise has been shown to halve breath-hold duration [27]. Furthermore, breath-hold duration may be shortened by disadvantageous conditions such as preexisting hypoxemia or hypercapnia [32,33]. Based on that rationale, any stressful condition that increases oxygen consumption, such as unresolved respiratory failure or any ongoing pathophysiological process that may interfere with weaning, will manifest itself in the form of shortened breath-hold duration.

Muscle strength is vital for the ability to hold a breath. During breath-holding, there is a constant central respiratory rhythm that is being voluntarily inhibited at the start of breath-holding and reappearing towards the breakpoint. Thus, in order for the breath hold to be sustained, there has to be a minimal muscle strength [14]. Considering these factors may explain why prolonged breath-holding actually reflects an optimal physiology whereby oxygen demand is in balance with supply, and CO_2_ production rate is matched by an equivalent ability to dispose of it.

During breath holding, respiratory drive may be represented by rhythmic negative pressure fluctuations and simultaneous rhythmic electromyography activity that appears and increases towards the breakpoint [28,34,35]. Breath-hold represents voluntarily ‘holding’ of the chest and suppressing expression of the central respiratory rhythm. Holding one’s breath entails the use of the respiratory muscles that need to overcome the inherent rhythmic activity of the respiratory drive. Therefore, we presume that any muscle weakness that may have developed during ICU stay in mechanically ventilated patients would result in a failure to perform a meaningful breath hold. 

Finally, the ability to hold a breath entails a normal neurological status and a full level of consciousness of the patient in order to execute a request as simple as holding one single breath. Hence, the ability to execute a simple request, such as holding a breath, the combination of a fully conscious patient with metabolic needs that do not overwhelm the patient’s ability to meet them, and the necessary muscle strength to perform the breath holding could result in a successful extubation.

In this study, after completing a SBT trial, patients, prior to extubation, were asked to hold their breath. It is known that breath-hold duration may almost be doubled by breath holding with hyperoxic gas mixtures, or by preceding breath-holding by voluntary or mechanical hyperventilation to lower PaCO_2_ levels. We did not perform hyperventilation or preoxygenation of the patients before asking them to hold their breath. Alternatively, breath-hold duration is almost halved by breath-holding from hypoxia or from hypercapnia, e.g., raising the inspired PCO_2_ to 65 mmHg [32,33].

Most patients who were not able to hold their breath eventually succumbed to recurrent respiratory failure and were reintubated. About 76% of patients who did not hold their breath were reintubated. This was independent of whether they had a successful SBT trial or not. Therefore, a successful or failed SBT trial could not predict or discern between patients who would fail or succeed in extubation. It is worth mentioning that patients who passed SBT had a similar reintubation rate to those who did not pass SBT; 24% and 23% of patients, respectively, were reintubated. Similar results were demonstrated in a study that examined extubation with or without prior SBT, in which they failed to demonstrate any advantage in performing SBT prior to extubation [36]. Successful extubation was achieved at similar rates in both groups.

In our study, we proved that adding the parameter of failure to hold a breath to standard SBT trials raised sensitivity. These findings support the notion that a simple bedside maneuver of breath holding could predict with 93% sensitivity a successful extubation, independently of a negative or positive SBT trial. The combination of both failures to pass SBT and failure to hold a breath carries a 79% chance of being reintubated. In addition, a successful SBT trial had a positive predictive value of only 75%, as compared to RBHI, which had a positive predictive value of 85%.

In this study the ROC curve of RBHI > 3 (sensitivity 75%, specificity 59%) demonstrated a stronger discriminative power compared to RSBI (sensitivity 47%, specificity 79%), with AUC of 0.723 (95% CI: 0.645–0.802) as compared to RSBI which had an AUC of 0.611 (95% CI: 0.526–0.697). This suggests that RBHI is more effective at predicting successful extubation outcomes than RSBI (Figure 3).

We showed that patients who failed SBT had a 42% chance of being reintubated, whereas patients who failed to hold their breath and had a RBHI less than 3 had a 90% chance of reintubation. Thus, a failed SBT trial was far less sensitive in predicting failure in extubation than RBHI, less than three and the inability to hold a breath.

This study had several limitations. Although the study recruited patients prospectively, the main comparison of breath-hold duration and RBHI was performed prospectively and was compared to the standard SBT prior to extubation. Furthermore, this study was noninterventional in nature. The breath-holding maneuver was concealed from the attending physician and was performed at the end of the SBT trial and just prior to extubation, and had no influence on the decision-making of the attending physician. Thus, patients were extubated regardless of the results of breath-holding, which was concealed from the attending physicians. Therefore, the breath-hold maneuver was compared only after the patient was extubated, and was compared prospectively to the results of the SBT trial. Lastly, this was a single-center study with all the limitations of a single center. Despite that, a cohort of 225 patients is large enough to identify differences between a breath-hold maneuver and a standard SBT, reaching statistical significance in favor of the breath-hold maneuver and RBHI as better predictors of successful extubation.

## 5. Conclusions

Our study adds a novel, yet relatively very simple bedside tool that, in addition to the standard SBT trial, may aid in increasing successful extubation rates. Higher RBHI, together with the ability to hold a breath in mechanically ventilated patients, may be superior to standard SBT. Hence, it may better predict a successful extubation. Conversely, the inability to hold a breath prior to extubation may better identify patients prone to weaning failure.

## Figures and Tables

**Table 1:** Patient’s characteristics and comparison between weaned and non-weaned mechanically ventilated patients.

Patient’s Characteristics	Successful Weaning (n = 171)	Failed Weaning (n = 54)	*p* Value (Total = 225)
Age, Mean ± SD	65 ± 17	73 ± 14	0.0015
Male (n, %)	94 (55)	32 (59)	0.6386
Female (n, %)	77 (45)	22 (41)	
BMI, Mean ± SD	28 ± 7	29 ± 9	0.9570
Use of NIV after extubation (n, %)	17 (10)	20 (37)	<0.0001
APACHE II score, Mean ± SD	19 ± 6	21 ± 6	0.0138
SOFA score on day of extubation, Mean ± SD	4 ± 2	4 ± 2	0.0143
Ventilator-free days, Mean ± SD	20 ± 8	6 ± 9	<0.0001
Number of failing organs at peak of disease, Mean ± SD	2 ± 1	3 ± 1	0.3200
Shock during ICU stay (n, %)	105 (61)	30 (56)	0.5242
ARDS during ICU stay (n, %)	30 (18)	8 (15)	0.8351
* VAP during ICU stay (n, %)	19 (11)	15 (28)	0.0046
Tracheostomy	0 (0)	16 (30)	<0.0001
ICU length of stay, Mean ± SD	9.67 (18)	20.54 (15)	<0.0001
ICU mortality (n, %)	2 (1)	10 (19)	<0.0001
Hospital mortality (n, %)	11 (6)	28 (52)	<0.0001

**SD**—standard deviation, **BMI**—body mass index, **NIV**—non-invasive ventilation, **SOFA**—The Sequential Organ Failure Assessment Score, **ICU**—intensive care unit, **ARDS**—acute respiratory stress disorder. * **VAP**—ventilatory acquired pneumonia during ICU stay prior to extubation.

**Table 2:** Respiratory parameters of weaned and non-weaned patients.

Respiratory Parameters	Successful Weaning (n = 171)	Failed Weaning (n = 54)	*p* Value (Total = 225)
Passed SBT, (n, %)	137 (80)	44 (82)	1.0000
RBHI, Mean ± SD	7 ± 5	4 ± 3	<0.0001
FVC, Mean ± SD	954 ± 386	761 ± 347	0.0002
Able to hold breath (n, %)	159 (93)	18 (33)	<0.0001
Breath-hold duration, Mean (sec) ± SD	7 ± 3	5 ± 3	<0.0001
PaO_2_/FiO_2_ ratio at the end of SBT trial, Mean ± SD	306 ± 101	308 ± 102	0.8998
RSBI Mean ± SD	51 ± 26	61± 28	0.0138
Length of mechanical ventilation prior to extubation, Mean ± SD	6 ± 7	9 ± 9	0.0044

**SBT**—spontaneous breathing trial, **RBHI**—Respiratory Breath Holding Index, **SD**—standard deviation, **FVC**—Forced Vital Capacity, **RSBI**—Rapid shallow breathing index. Measured as respiratory rate/tidal volume (RR/TV) in liters.

**Table 3:** Characteristics of patients who passed the Spontaneous Breathing Trial.

Characteristics	Passed SBT and Were Able to Hold Breath (n = 145)	Passed SBT, But Did Not Hold Breath (n = 36)	Total (n = 181)	*p* Value
Successfully Extubated (n, %)	129 (89)	8 (24)	137 (76)	<0.0001
Length of Mechanical Ventilation prior to Extubation, Mean (days) ± SD	6 ± 7	10 ± 9	7 ± 8	0.0005
ICU Length of Stay, Mean (days) ± SD	9 ± 9	18 ± 13	11 ± 11	<0.0001
Hospital Length of Stay, Mean (days) ± SD	24 ± 19	43 ± 29	27 ± 23	<0.0001
ICU Mortality (n, %)	6 (4)	4 (11)		0.1129
Hospital Mortality (n, %)	14 (10)	17 (47)		<0.0001

**SBT**—spontaneous breathing trial, **ICU**—intensive care unit.

**Table 4:** Predictors for successful extubation.

Predictors	Sensitivity	Specificity	PPV	NPV	Cut Off (Sec)
SBT	0.80	0.19	0.757	0.227	No cutoff
Ability to hold breath	0.93	0.66	0.898	0.75	No cutoff
breath-hold duration	0.72	0.61	0.855	0.412	4.5
RBHI	0.75	0.59	0.853	0.433	3.4
RSBI	0.47	0.78	0.87	0.316	43

**PPV**—positive predictive value, **NPP**—negative predictive value, **SBT**—spontaneous breathing trial, **RBHI**—Respiratory Breath Holding Index, **RSBI**—Rapid shallow breathing index. Measured as respiratory rate/tidal volume (RR/TV) in liters.

## Data Availability

All data generated or analysed during this study are included in this published article. The datasets used and/or analysed during the current study are available from the corresponding author on reasonable request.

## Abbreviations

The following abbreviations are used in this manuscript: Area under the curve (AUC), forced vital capacity (FVC), Glasgow coma scale (GCS), intensive care unit (ICU), positive end-expiratory pressure (PEEP), rapid shallow breath index (RSBI), Respiratory Breath Hold Index (RBHI), sequential organ failure assessment (SOFA), spontaneous breathing trial (SBT), ventilated acquired pneumonia (VAP).

## References

[1] Pham T., Heunks L. (2023). Weaning from mechanical ventilation in intensive care units across 50 countries (WEAN SAFE): A multicentre, prospective, observational cohort study. Lancet Respir. Med..

[2] Frutos-Vivar F., Esteban A. (2011). Outcome of reintubated patients after scheduled extubation. J. Crit. Care.

[3] Thille A.W., Harrois A. (2011). Outcomes of extubation failure in medical intensive care unit patients. Crit. Care Med..

[4] De Lassence A., Alberti C. (2002). Impact of unplanned extubation and reintubation after weaning on nosocomial pneumonia risk in the intensive care unit: A prospective multicenter study. Anesthesiology.

[5] Ding X., Ma X. (2022). Effect of ICU quality control indicators on VAP incidence rate and mortality: A retrospective study of 1267 hospitals in China. Crit. Care.

[6] Gao F., Yang L.H. (2016). The effect of reintubation on ventilator-associated pneumonia and mortality among mechanically ventilated patients with intubation: A systematic review and meta-analysis. Heart Lung.

[7] Xie J., Cheng G. (2019). To extubate or not to extubate: Risk factors for extubation failure and deterioration with further mechanical ventilation. J. Card. Surg..

[8] Gershengorn H.B., Scales D. (2016). Association between overnight extubations and outcomes in the intensive care unit. JAMA Intern. Med..

[9] Ionescu F., Zimmer M.S. (2021). Extubation failure in critically ill COVID-19 patients: Risk factors and impact on in-hospital mortality. J. Intensive Care Med..

[10] Menon N., Joffe A.M. (2012). Occurrence and complications of tracheal reintubation in critically ill adults. Respir. Care.

[11] Gowardman J.R., Huntington D. (2006). The effect of extubation failure on outcome in a multidisciplinary Australian intensive care unit. Crit. Care Resusc..

[12] Béduneau G., Pham T. (2017). Epidemiology of Weaning Outcome according to a New Definition. The WIND Study. Am. J. Respir. Crit. Care Med..

[13] Dettmer M.R., Damuth E. (2017). Prognostic factors for long-term mortality in critically ill patients treated with prolonged mechanical ventilation: A systematic review. Crit. Care Med..

[14] Brown C.V., Daigle J.B. (2011). Risk factors associated with early reintubation in trauma patients: A prospective observational study. J. Trauma Acute Care Surg..

[15] Piriyapatsom A., Williams E.C. (2016). Prospective observational study of predictors of re-intubation following extubation in the surgical ICU. Respir. Care.

[16] Torrini F., Gendreau S. (2021). Prediction of extubation outcome in critically ill patients: A systematic review and meta-analysis. Crit. Care.

[17] Thille A.W., Boissier F. (2015). Risk factors for and prediction by caregivers of extubation failure in ICU patients: A prospective study. Crit. Care Med..

[18] Menguy J., De Longeaux K. (2023). Defining predictors for successful mechanical ventilation weaning, using a data-mining process and artificial intelligence. Sci. Rep..

[19] Baptistella A.R., Mantelli L.M. (2021). Prediction of extubation outcome in mechanically ventilated patients: Development and validation of the Extubation Predictive Score (ExPreS). PLoS ONE.

[20] Rojek-Jarmuła A., Hombach R. (2017). APACHE II score cannot predict successful weaning from prolonged mechanical ventilation. Chron. Respir. Dis..

[21] Trivedi V., Chaudhuri D. (2022). The usefulness of the rapid shallow breathing index in predicting successful extubation: A systematic review and meta-analysis. Chest.

[22] Jia D., Wang H. (2024). Rapid shallow breathing index predicting extubation outcomes: A systematic review and meta-analysis. Intensive Crit. Care Nurs..

[23] Peñuelas O., Frutos-Vivar F. (2011). Characteristics and outcomes of ventilated patients according to time to liberation from mechanical ventilation. Am. J. Respir. Crit. Care Med..

[24] Li W., Zhang Y. (2023). The risk factors of reintubation in intensive care unit patients on mechanical ventilation: A systematic review and meta-analysis. Intensive Crit. Care Nurs..

[25] Miu T., Joffe A.M. (2014). Predictors of reintubation in critically ill patients. Respir. Care.

[26] Rodbard S. (1947). The effect of oxygen, altitude and exercise on breath-holding time. Am. J. Physiol..

[27] Cummings E.G. (1962). Breath holding at beginning of exercise. J. Appl. Physiol..

[28] Lin Y.C., Lally D.A. (1974). Physiological and conventional breath-hold break points. J. Appl. Physiol..

[29] Ward S.A., Macias D. (2001). Is breath-hold time an objective index of exertional dyspnoea in humans. Eur. J. Appl. Physiol..

[30] Stevens C.D., Ferris E.B. (1946). Voluntary breath holding II. Pulmonary gas exchange during breath holding. J. Clin. Investig..

[31] Hong S.K., Lin Y.C. (1975). Alveolar gas exchanges and cardiovascular functions during breath holding with air. J. Appl. Physiol..

[32] Godfrey S., Campbell E.J.M. (1969). Mechanical and chemical control of breath holding. Q. J. Exp. Physiol. Cogn. Med. Sci. Transl. Integr..

[33] Kelman G.R., Wann K.T. (1971). Mechanical and chemical control of breath holding. Q. J. Exp. Physiol..

[34] Whitelaw W.A., McBride B. (1981). Respiratory neuromuscular output during breath holding. J. Appl. Physiol..

[35] Whitelaw W.A., McBride B. (1987). Effect of lung volume on breath holding. J. Appl. Physiol..

[36] Wang J., Ma Y., Fang Q. (2013). Extubation with or without spontaneous breathing trial. Crit. Care Nurse.

